# Nationwide Characteristics of Patients Admitted to Hospital for the First Time Due to Primary Liver Cancer and Intrahepatic Bile Duct Cancer in Poland in 2012–2023

**DOI:** 10.3390/cancers18162579

**Published:** 2026-08-11

**Authors:** Agnieszka Genowska, Jerzy Jaroszewicz, Dorota Zarębska-Michaluk, Katarzyna Lewtak, Krystyna Dobrowolska, Krzysztof Kanecki, Anna Parfieniuk-Kowerda, Aneta Nitsch-Osuch, Piotr Rzymski, Robert Flisiak

**Affiliations:** 1 Department of Public Health, Medical University of Bialystok, 15-295 Bialystok, Poland; 2 Department of Infectious Diseases and Hepatology, Medical University of Silesia, 40-055 Katowice, Poland; jerzyjr@gmail.com; 3 Department of Infectious Diseases and Allergology, Jan Kochanowski University, 25-317 Kielce, Poland; dorota1010@tlen.pl; 4 Department of Social Medicine and Public Health, Medical University of Warsaw, 00-106 Warsaw, Poland; katarzyna.lewtak@wum.edu.pl (K.L.); krzysztof.kanecki@wum.edu.pl (K.K.); aneta.nitsch-osuch@wum.edu.pl (A.N.-O.); 5 Collegium Medicum, Jan Kochanowski University, 25-317 Kielce, Poland; 6 Department of Infectious Diseases and Hepatology, Medical University of Bialystok, 15-540 Bialystok, Poland; anna.parfieniuk-kowerda@umb.edu.pl (A.P.-K.); robert.flisiak1@gmail.com (R.F.); 7 Department of Environmental Medicine, Poznan University of Medical Sciences, 60-806 Poznan, Poland; rzymskipiotr@gmail.com

**Keywords:** liver neoplasms, hepatocellular carcinoma, cholangiocarcinoma, hospitalization, epidemiology, Poland

## Abstract

Background/Objectives: Primary liver and intrahepatic bile duct cancers remain important causes of cancer morbidity and mortality worldwide, yet contemporary nationwide data on affected populations in Poland are limited. This study evaluated sociodemographic patterns of first-time hospitalizations due to malignant neoplasms of the liver and intrahepatic bile ducts in Poland during 2012–2023. Methods: Data were obtained from the Nationwide General Hospital Morbidity Study and included 30,898 first-time hospitalizations identified using the ICD-10 category C22. First-time hospitalization rates were assessed according to sex, age, place of residence, disease subtype, length of stay, and comorbidities. Results: Men accounted for 57.7% of urban and 58.4% of rural cases and had significantly higher first-time hospitalization rates than women. These rates were higher among urban than rural residents (men: 9.5 vs. 6.0 per 100,000; women: 6.3 vs. 4.2 per 100,000). Liver cell carcinoma was the most frequent subtype (36.3%), followed by intrahepatic bile duct carcinoma (12.6%). Hospitalizations were uncommon before the age of 40 years and peaked in the 65–69-year age group. Mean age increased over time among men and urban women. In 2020, the number of first-time hospitalizations decreased from 2754 to 2060, corresponding to 694 fewer hospitalizations and a 25.2% decline compared with 2019. This decrease occurred against the background of nationwide reductions of 22.1% in patients treated in general-hospital wards and 8.1% in oncology-ward patients, suggesting that pandemic-related healthcare disruption may have contributed to the observed decline. Hospitalization rates subsequently increased. Rates for liver cell carcinoma remained relatively stable in most population groups, whereas those for intrahepatic bile duct carcinoma were significantly higher in 2020–2023 than in 2012–2019 across all sex and place-of-residence groups. Length-of-stay patterns were broadly similar across the analyzed groups, and diseases of the digestive system were the most common comorbidity category (23.1%). Conclusions: The findings demonstrate important sociodemographic differences and temporal changes in first-time hospitalizations due to malignant neoplasms of the liver and intrahepatic bile ducts in Poland. These findings suggest evolving epidemiological patterns and underscore the need for continued prevention, surveillance, and early detection efforts.

## Simple Summary

Primary liver cancer is one of the leading causes of cancer-related illness and death worldwide. Understanding who is affected and how the disease changes over time is essential for improving prevention, early diagnosis, and treatment. However, recent nationwide information for Poland has been limited. This study examined first-time hospitalizations for liver cancer and cancer of the bile ducts within the liver in Poland between 2012 and 2023. We analyzed differences by age, sex, place of residence, disease type, length of hospital stay, and other health conditions. The findings show important differences between men and women, urban and rural populations, and changes in the types of liver cancer diagnosed over time. These results provide updated evidence on the burden of liver cancer in Poland and may help researchers, healthcare professionals, and policymakers improve disease surveillance, prevention strategies, and early detection programs.

## 1. Introduction

Primary liver cancer is among the most important causes of cancer-related morbidity and mortality worldwide [1]. According to recent global estimates, liver cancer ranks among the leading causes of cancer death and remains a major public health challenge due to its poor prognosis and frequent late diagnosis [2,3]. Hepatocellular carcinoma (HCC) accounts for approximately 75–85% of all primary liver cancers [4], while intrahepatic cholangiocarcinoma and several rarer histological subtypes constitute the remaining cases [5]. The preferred treatment selection is primarily guided by the Barcelona Clinic Liver Cancer strategy, based on the tumor stage established by imaging studies, liver function, and patient performance status. Potentially curative approaches include surgical partial hepatectomy, liver transplantation, as well as locoregional treatment by radiofrequency ablation or external beam radiation therapy in very early and early stages of HCC. In intermediate and advanced disease, locoregional procedures, particularly variants of intra-arterial embolizing therapies, or systemic treatment with tyrosine kinase inhibitors, anti-VEGF antibodies, and immune checkpoint inhibitor-based combinations are recommended [6,7]. Despite advances in surveillance, diagnostic imaging, and treatment modalities, long-term survival remains limited, particularly among patients diagnosed at advanced stages of disease [8,9].

The epidemiology of liver cancer varies considerably across geographical regions, reflecting differences in the prevalence of underlying risk factors [1,10]. Chronic infection with the hepatitis B virus (HBV) and hepatitis C virus (HCV) has historically been responsible for the majority of HCC cases worldwide [11]. However, in many high-income countries, including those in Europe, the etiological landscape is changing. The burden attributable to viral hepatitis has gradually declined owing to vaccination programs, improved blood safety, and the availability of highly effective antiviral therapies [12,13]. Simultaneously, the contribution of metabolic dysfunction-associated steatotic liver disease (MASLD), obesity, type 2 diabetes mellitus, and alcohol-related liver disease has increased, contributing to a growing number of patients at risk of developing HCC [14,15,16,17]. Population aging further influences disease occurrence, as the incidence of liver cancer rises substantially with age [18].

Marked differences in liver cancer incidence and mortality are observed according to sex and socioeconomic characteristics. Men are affected two to four times more frequently than women, a phenomenon attributed to differences in exposure to risk factors, hormonal influences, and biological mechanisms involved in hepatocarcinogenesis [19,20]. Socioeconomic status, healthcare accessibility, educational attainment, and place of residence may also affect the risk of disease development, participation in surveillance programs, timeliness of diagnosis, and clinical outcomes. Urban–rural disparities have been reported in several countries, although their direction and magnitude vary depending on local healthcare systems and population characteristics [21,22].

In Poland, liver cancer remains a significant health concern. Previous epidemiological analyses have demonstrated a substantial disease burden and persistently high mortality associated with primary liver malignancies [23,24]. At the same time, demographic changes, shifts in the prevalence of chronic liver diseases, increasing rates of metabolic disorders, and evolving healthcare practices may have altered the characteristics of patients presenting with liver cancer. Nevertheless, comprehensive nationwide assessments of sociodemographic patterns among newly hospitalized patients with liver malignancies remain limited. Such analysis may provide valuable insights into populations at greatest risk, help identify temporal changes in disease occurrence, and support planning of preventive, diagnostic, and therapeutic strategies.

Hospitalization databases constitute an important source of epidemiological information, particularly for diseases that frequently require specialist inpatient management [25]. Analyses of first-time hospitalizations approximate newly recognized cases entering the healthcare system and enable assessment of demographic and geographic patterns at the population level, though some patients may be diagnosed and managed in outpatient settings [26,27,28]. Long-term monitoring of these indicators may also reveal the effects of changing risk-factor profiles, public health interventions, and healthcare accessibility [25].

Therefore, this study aimed to analyze the sociodemographic characteristics of patients hospitalized for the first time due to hepatocellular carcinoma and other primary malignant neoplasms of the liver and intrahepatic bile ducts in Poland during the period 2012–2023 and to evaluate temporal trends according to sex, age, place of residence, and disease subtype.

## 2. Materials and Methods

### 2.1. Study Design

For this study, analyses were limited to first-time hospitalizations between January 2012 and December 2023 among patients diagnosed with malignant neoplasm of the liver and intrahepatic bile ducts. The study group included 30,898 first-time hospitalization cases out of a total of 100,904. First-time hospital admissions were identified using sociodemographic data, principal diagnoses, admission and discharge dates, and comorbidities. Hospitalization cases were identified using ICD-10 codes recorded as the principal or related/significantly associated diagnosis: C22 (malignant neoplasm of liver and intrahepatic bile ducts), C22.0 (liver cell carcinoma), C22.1 (intrahepatic bile duct carcinoma), and C22.7 (other specified carcinomas of liver) and other malignant neoplasms (hepatoblastoma C22.2, angiosarcoma of liver C22.3, other sarcomas of liver C22.4, liver unspecified C22.9). Comorbidities were categorized according to ICD-10 chapters, including certain infectious and parasitic diseases (A00–B99), neoplasms (C00–D48), diseases of the blood and blood-forming organs and certain disorders involving the immune mechanism (D50–D89), endocrine, nutritional and metabolic diseases (E00–E90), diseases of the circulatory system (I00–I99), diseases of the respiratory system (J00–J99), diseases of the digestive system (K00–K93), diseases of the genitourinary system (N00–N99), symptoms, signs and abnormal clinical and laboratory findings, not elsewhere classified (R00–R99), factors influencing health status and contact with health services (Z00–Z99).

The study was conducted in accordance with the Declaration of Helsinki, and its protocol was approved by the Ethics Committee of the Medical University of Bialystok (approval number APK-002-149-2024, dated 22 February 2024).

### 2.2. Data Source

Data were obtained from the Nationwide General Hospital Morbidity Study (NGHMS), the principal national source of individual-level hospital morbidity data in Poland, conducted by the National Institute of Public Health NIH—National Research Institute (NIPH NIH-NRI) within the Program of Statistical Surveys of Official Statistics. The NGHMS collects data from both public and private hospitals (excluding psychiatric wards) and covers all patients admitted to the hospital, regardless of their place of residence or insurance status. All hospitalization cases recorded in the main ledger (the principal register of admissions and discharges), including both day cases and longer inpatient stays, are subject to reporting [29].

The collected data enable a comprehensive analysis of the structure and frequency of hospitalizations. In this study, the following data were collected for each patient:

- Patient sociodemographic characteristics, such as age, sex, and place of residence;
- Principal diagnosis (the reason behind the patient’s hospitalization) and comorbidities relevant to the course of hospitalization coded according to the International Statistical Classification of Diseases and Related Health Problems, 10th Revision (ICD-10);
- Information on hospitalization: admission and discharge dates, types of admission and discharge.

This study did not involve patient participation. All data extracted from the NGHMS database were anonymized and did not contain any information that could identify patients.

### 2.3. Statistical Analyses

Quantitative variables were described using the number of patients hospitalized with a malignant neoplasm of the liver and intrahepatic bile ducts, mean ± standard deviation, and median with interquartile range (IQR). Categorical variables were described as percentages of patients per category. Patients were categorized by sociodemographic data (age, sex, date of birth, place of residence) and grouped by hospital length of stay (LOS) (0–3 days, 4–7 days, ≥8 days) [30]. The calculated data of included (1) the number and % of first-time hospitalizations due to diagnoses with malignant neoplasm of liver and intrahepatic bile ducts and rates per 100,000 population per year: total, by sex, and place of residence for 2012–2023, (2) mean and median age of hospitalizations due to malignant neoplasm of liver and intrahepatic bile ducts in 2012–2023 (sex and place of residence), (3) first-time hospitalization rate due to malignant neoplasm of liver and intrahepatic bile ducts per 100,000 population per year by sex and place of residence for 2012–2023, (4) percentage of first-time hospitalizations due to malignant neoplasm of liver and intrahepatic bile ducts in LOS classes (0–3 days, 4–7 days, ≥8 days), (5) percentage of comorbid diagnoses among patients hospitalized for the first time due to diagnoses with malignant neoplasm of liver and intrahepatic bile ducts. To calculate the annual first-time hospitalization rates due to malignant neoplasms of the liver and intrahepatic bile ducts per 100,000 population between 2012 and 2023, annual population data for Poland, obtained from Statistics Poland, were used as the denominator. In addition, age-standardized hospitalization rates (ASHRs) per 100,000 population were calculated using the direct standardization method with the 2013 European Standard Population as the reference population, separately for men and women and for each calendar year.

Temporal trends in first-time hospitalizations due to malignant neoplasms of the liver and intrahepatic bile ducts were evaluated according to demographic characteristics using simple linear regression, with calendar year entered as the independent variable. Statistical significance of the regression slope was assessed using a two-sided *t*-test. Regression coefficients (β), standard errors (SE), 95% confidence intervals (95% CI), and *p* values were reported, while the coefficient of determination (R^2^) was used to assess the goodness of fit of each model. Statistical significance was set at *p* < 0.05. Analyses were performed using Statistica version 13 (TIBCO Software Inc., Palo Alto, CA, USA) and WinPepi version 11.65 [31].

## 3. Results

From 2012 to 2023, a total of 30,898 first-time hospitalizations due to malignant neoplasms of the liver and intrahepatic bile ducts were recorded in Poland. Men accounted for a greater proportion of first-time hospitalizations, representing 57.7% of all hospitalizations among urban residents and 58.4% among rural residents. During the study period, the first-time hospitalization rates differed significantly between urban residents (men: 9.5 per 10^5^ vs. women: 6.3 per 10^5^) and rural residents (men: 6.0 per 10^5^ vs. women: 4.2 per 10^5^). The mean age-standardized hospitalization rate (ASHR) for first-time hospitalizations due to malignant neoplasms of the liver and intrahepatic bile ducts was 9.9 per 10^5^ among men and 5.4 per 10^5^ among women, with statistically significant differences between the sexes (*p* < 0.001) (Table 1).

**Figure 1:**
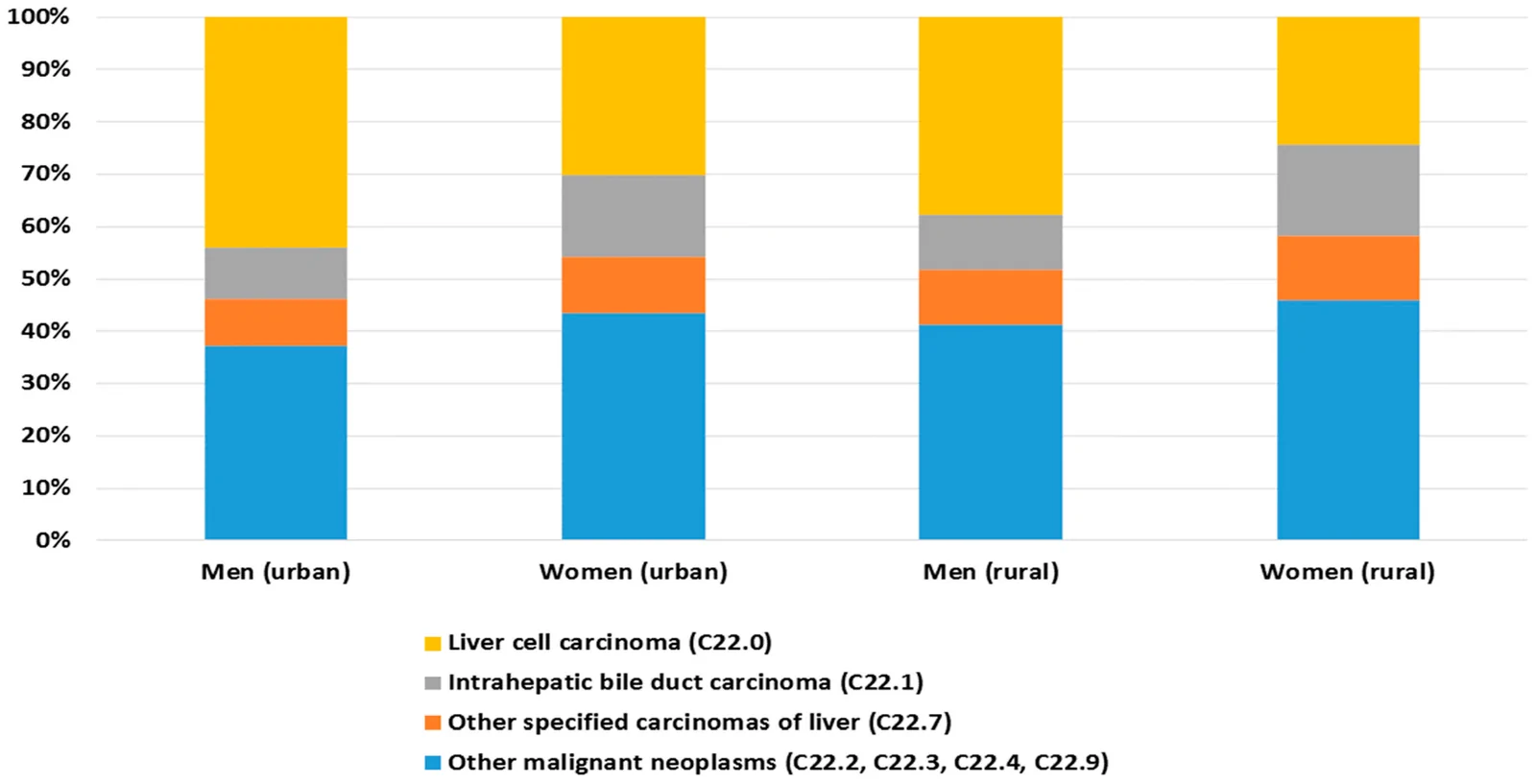
Structure of first-time hospitalization cases due to malignant neoplasms of the liver and intrahepatic bile ducts by subtype, sex, and place of residence (2012–2023).

Among the analyzed diagnostic categories, liver cell carcinoma was the most common, accounting for 36.3% of all first-time hospitalizations, followed by intrahepatic bile duct carcinoma (12.6%) and other specified carcinomas of the liver (10.1%). Distinct sex- and residence-related differences were observed in the distribution of liver and intrahepatic bile duct cancer subtypes (Figure 1). Liver cell carcinoma was most frequently observed among men living in urban areas (44.0%). Intrahepatic bile duct carcinoma showed the highest proportion among rural women (17.4%), while other specified carcinomas of the liver were also most common in rural women, accounting for 12.4% of cases.

**Figure 2:**
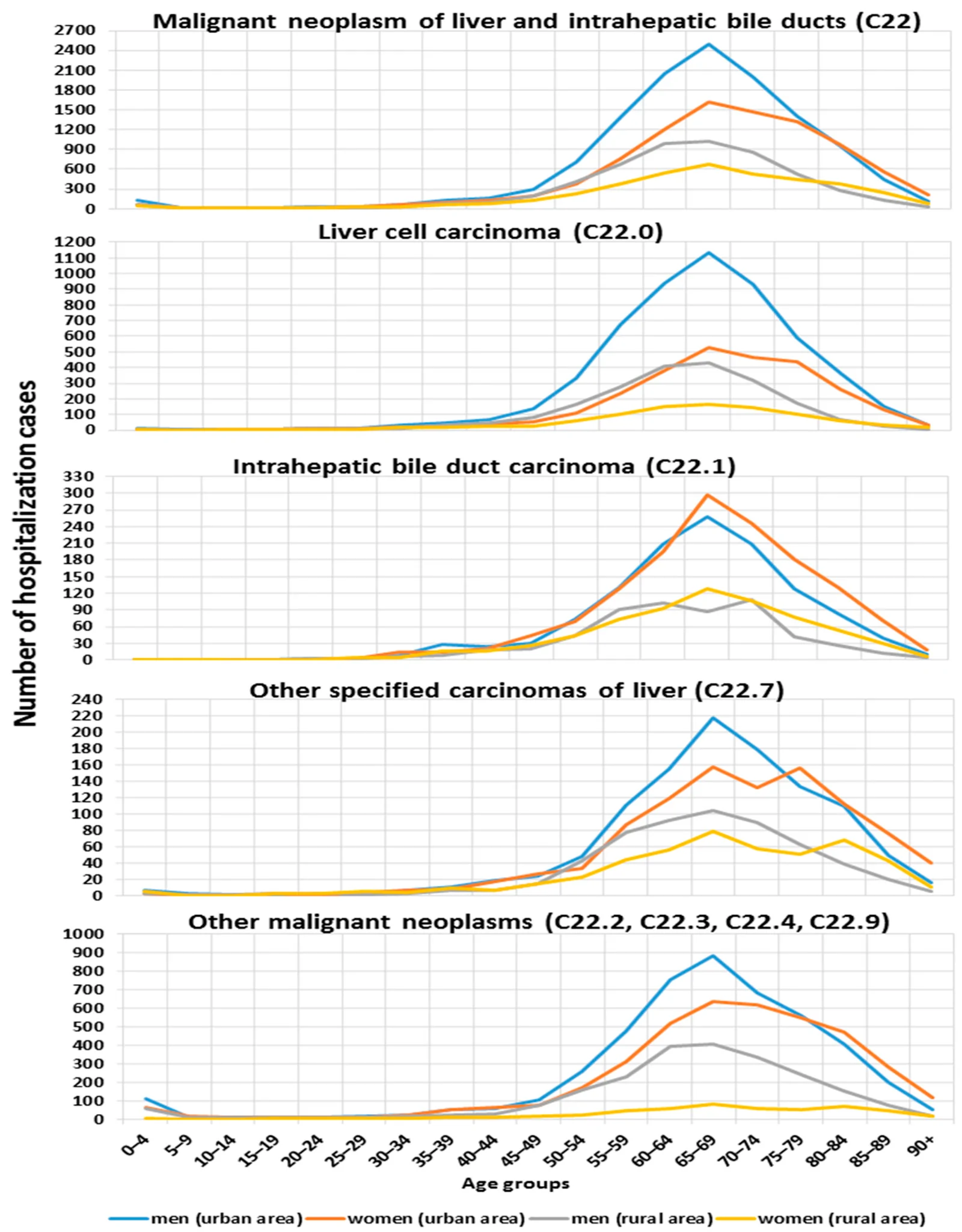
First-time hospitalization cases of malignant neoplasms of the liver and intrahepatic bile ducts by age groups, sex, and place of residence (2012–2023).

Age was associated with the distribution of first-time hospitalizations due to malignant neoplasms of the liver and intrahepatic bile ducts (Figure 2). First-time hospitalizations were uncommon among individuals younger than 40 years. The number of cases increased substantially between 40 and 69 years, peaking in the 65–69-year age group. At this age, the hospitalization rate ranged from 17.3% among rural women to 20.0% among urban men. Subsequently, the number of hospitalizations declined in individuals aged 70 years and older.

The mean age of patients increased significantly over the study period (β = 0.149, SE = 0.045, 95% CI: 0.049–0.249; *p* = 0.008, R^2^ = 0.52). Similar increasing trends were observed among urban men (β = 0.109, SE = 0.041, 95% CI: 0.019–0.199; *p* = 0.023, R^2^ = 0.42), rural men (β = 0.247, SE = 0.085, 95% CI: 0.056–0.437; *p* = 0.016, R^2^ = 0.45), and urban women (β = 0.181, SE = 0.073, 95% CI: 0.019–0.344; *p* = 0.032, R^2^ = 0.38), whereas no significant temporal trend was identified among rural women (Table 2).

The age of patients who were first-time hospitalized due to malignant neoplasms of the liver and intrahepatic bile ducts varied according to sex and place of residence. Throughout the study period, women were generally older than men, while urban residents tended to be older than rural residents. The highest mean ages were observed among urban women, ranging from 65.6 years in 2012 to 69.7 years in 2019, whereas the lowest values were recorded among rural men, ranging from 61.0 to 65.8 years.

This upward trend was interrupted after 2019, when a decline in mean age was observed in 2020 across all population groups. In subsequent years, mean age values increased gradually but generally remained below or close to the peak levels observed before the COVID-19 pandemic.

Median age showed a similar pattern, increasing over time in most study groups and reaching the highest values between 2019 and 2021. Across all years, interquartile ranges indicated that the majority of first-time hospitalized patients were between approximately 58 and 78 years of age. Overall, urban residents were significantly older than rural residents among both men (65.9 vs. 63.7 years; *p* < 0.001) and women (68.2 vs. 66.3 years; *p* < 0.001). Furthermore, women were significantly older than men in both urban and rural areas (*p* < 0.001 for both comparisons) (Table 2).

**Figure 3:**
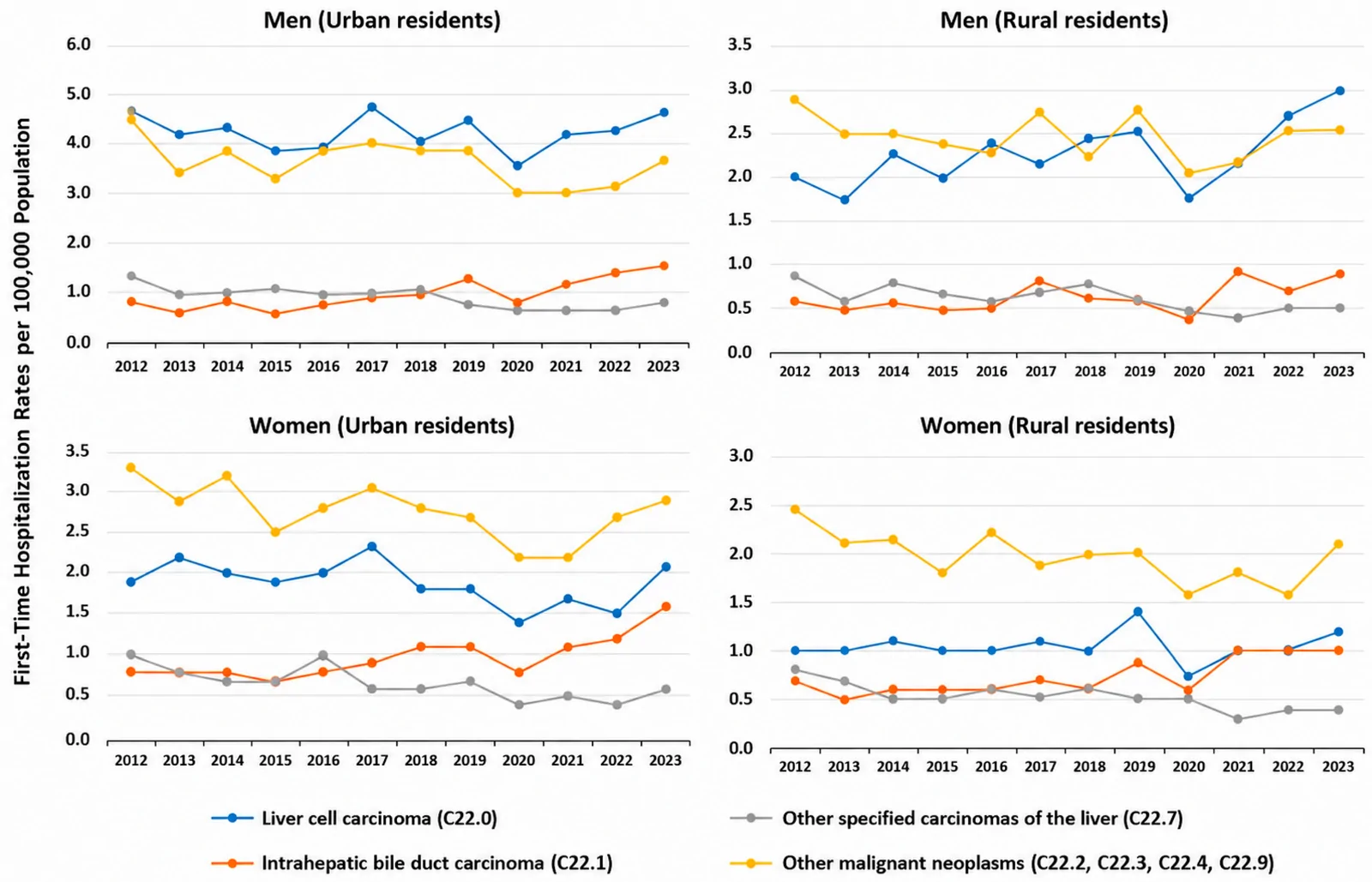
First-time hospitalization rates due to malignant neoplasms of the liver and intrahepatic bile ducts by sex and place of residence, Poland, 2012–2023.

Throughout the study period, first-time hospitalization rates for all analyzed subtypes of malignant neoplasms of the liver and intrahepatic bile ducts were generally higher among urban than rural residents and among men than women. In the first year of the COVID-19 pandemic (2020), first-time hospitalization rates declined from 0% to 50% compared with 2019 across all analyzed diagnostic categories. However, in the subsequent pandemic years, increasing trends were observed for most analyzed malignant neoplasms. For intrahepatic bile duct carcinoma, first-time hospitalization rates in 2023 were approximately 67% to 125% higher than those recorded in 2020 across the four sex- and place-of-residence groups (Figure 3). Consistent with these observations, linear regression analysis demonstrated a significant increasing temporal trend for intrahepatic bile duct carcinoma (β = 0.047, SE = 0.010, 95% CI: 0.025–0.070; *p* < 0.001, R^2^ = 0.68). In contrast, significant decreasing trends were observed for other specified carcinomas of the liver (β = −0.038, SE = 0.007, 95% CI: −0.052 to −0.023; *p* < 0.001, R^2^ = 0.76) and other malignant neoplasms of the liver and intrahepatic bile ducts (β = −0.050, SE = 0.022, 95% CI: −0.098 to −0.001; *p* < 0.05, R^2^ = 0.34).

Analysis of annual trends in first-time hospitalization rates showed no significant changes in the study group. However, a significantly increasing trend in the first-time hospitalization rates was observed for intrahepatic bile duct carcinoma (*p* < 0.001, R^2^ = 0.68), whereas significantly decreasing trends were observed for other specified carcinomas of the liver (*p* < 0.01, R^2^ = 0.76) and other malignant neoplasms of the liver and intrahepatic bile ducts (*p* < 0.05, R^2^ = 0.34).

A comparison of the pre-pandemic (2012–2019) and pandemic (2020–2023) periods revealed substantial changes in first-time hospitalization rates. Mean first-time hospitalization rates were generally significantly higher during the pre-pandemic period than during the pandemic period for most diagnostic categories. The only exceptions were intrahepatic bile duct carcinoma in both sexes and places of residence, and liver cell carcinoma among rural men, for which first-time hospitalization rates were significantly higher during the pandemic period than during the pre-pandemic period (*p* < 0.005) (Table 3).

The distribution of first-time hospitalizations due to malignant neoplasms of the liver and intrahepatic bile ducts according to length of stay (LOS) was generally similar across sex and place-of-residence groups (Table 4). For most diagnostic categories, hospitalizations lasting zero to three days and those lasting eight days or longer accounted for the largest proportions of cases, whereas stays of four to seven days were less frequent.

Among patients with liver cell carcinoma, approximately 40% of hospitalizations lasted zero to three days and 36–42% lasted at least eight days, with comparable distributions observed among urban and rural residents. A similar pattern was found for intrahepatic bile duct carcinoma, although short hospitalizations were more common, accounting for nearly half of all admissions in both sexes and places of residence.

For other specified carcinomas of the liver, prolonged hospitalizations (LOS ≥ eight days) represented the largest proportion of cases, accounting for approximately one-half of all admissions among both men and women. Likewise, among patients with other malignant neoplasms of the liver and intrahepatic bile ducts, hospitalizations lasting at least eight days constituted the most frequent LOS category.

Overall, only a few significant differences in LOS distribution were observed between urban and rural residents. The only statistically significant difference was observed among men hospitalized due to other malignant neoplasms of the liver and intrahepatic bile ducts, for whom hospitalizations lasting four to seven days were more common among rural than urban residents (*p* < 0.05).

**Figure 4:**
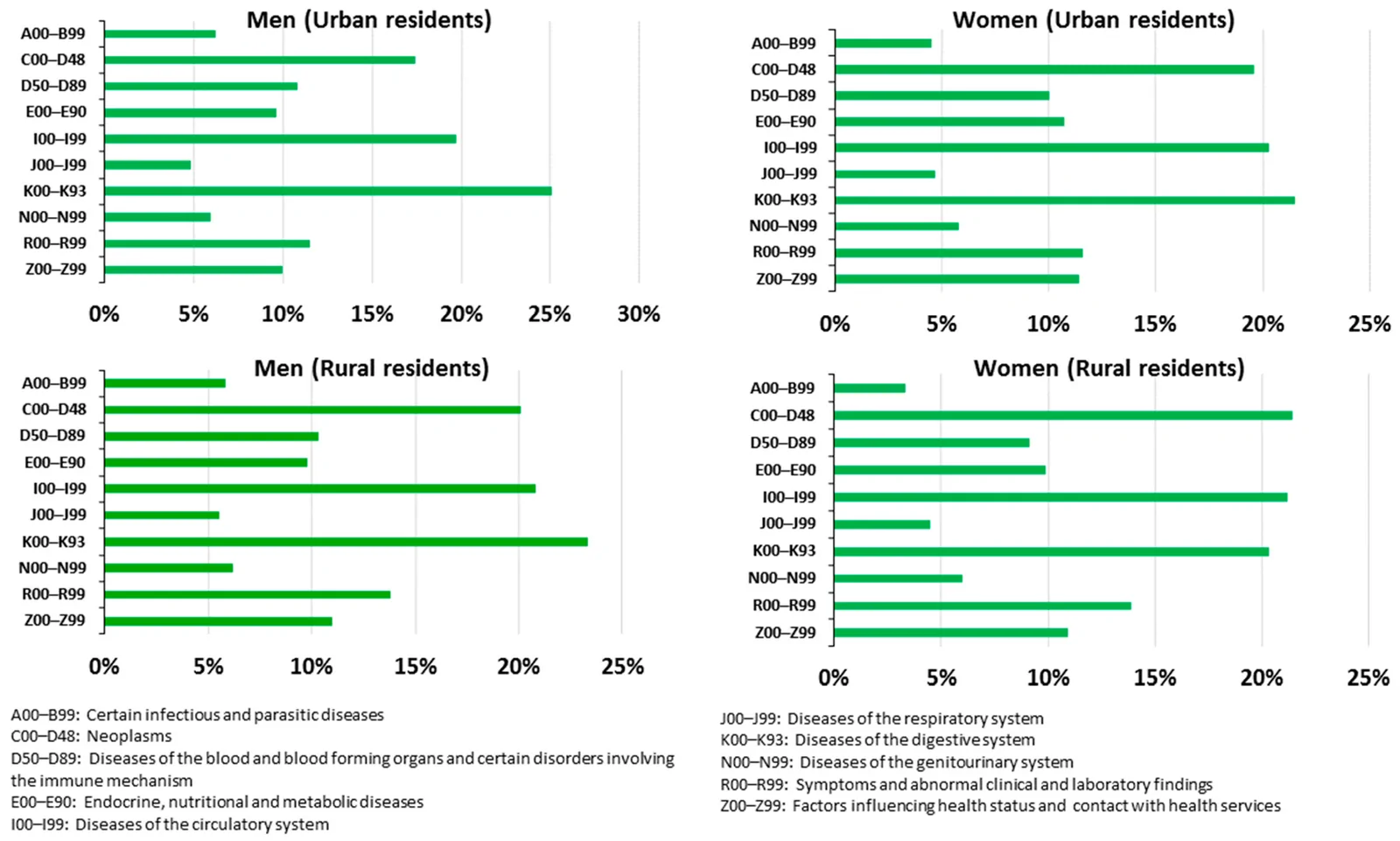
The most frequent ICD-10 comorbidity categories among patients first-time hospitalized due to malignant neoplasms of the liver and intrahepatic bile ducts according to sex and place of residence, Poland, 2012–2023.

Figure 4 presents the distribution of the most frequent ICD-10 comorbidity categories among patients first-time hospitalized due to malignant neoplasms of the liver and intrahepatic bile ducts, stratified by sex and place of residence. Diseases of the digestive system, diseases of the circulatory system, and neoplasms were the three most common comorbidity categories in all analyzed subgroups. Diseases of the digestive system were consistently more frequent among men than women in both urban (25.1% vs. 21.5%) and rural residents (23.3% vs. 20.3%). In contrast, neoplasms were more common among women than men in both urban (19.6% vs. 17.4%) and rural populations (21.4% vs. 20.1%). Likewise, diseases of the circulatory system occurred slightly more frequently among women than men, with proportions of 20.3% versus 19.7% among urban residents and 21.2% versus 20.8% among rural residents. Rural residents generally showed higher proportions of diseases of the circulatory, respiratory, genitourinary, and symptom-related (R00–R99) categories than the corresponding urban residents, whereas the overall distribution of the remaining comorbidity categories was comparable across the analyzed groups.

## 4. Discussion

This nationwide study characterizes first-time hospitalizations due to malignant neoplasms of the liver and intrahepatic bile ducts in Poland during 2012–2023. Rather than estimating cancer incidence, it describes the population entering inpatient or day-hospital pathways, thereby identifying demographic, geographic, and temporal patterns relevant to healthcare planning. The findings should be interpreted in the context of the growing global burden of liver cancer, with population-level five-year survival remaining approximately 20% or lower, and recurrence occurring in up to 70% of patients [32,33]. Recent projections suggest that annual incident cases may increase from approximately 870,000 in 2022 to more than 1.5 million by 2050 if current trends continue [34,35]. At the same time, a substantial proportion of liver cancers remains preventable through effective control of major risk factors, including chronic hepatitis B and C virus infection, harmful alcohol consumption, and MASLD. Estimates suggest that modifying these risk factors could avert 7.7–15.1 million deaths from hepatic malignancies by 2050 [36,37]. Against this background, the present findings provide insight not only into the scale of the hospitalization burden in Poland but also into which population groups are most frequently reaching hospital-based care and how these patterns have changed over time.

The predominance of men observed in our study is consistent with findings reported worldwide. Men represented nearly 60% of all first-time hospitalizations and exhibited markedly higher hospitalization rates than women, irrespective of place of residence. This persistent difference may reflect the cumulative effects of sex-specific exposure, biological susceptibility, and healthcare utilization rather than a single dominant mechanism. Historically, men have experienced a higher prevalence of chronic viral hepatitis, alcohol-related liver disease, smoking, and other behavioral risk factors [38,39]. Obesity, diabetes, adipokines, and body composition show sex-specific associations with liver cancer, with some risk patterns being more pronounced in males [40,41]. Biological mechanisms may also contribute, including the protective effects of estrogens and the influence of androgen-related signaling pathways on hepatocarcinogenesis [42,43].

A less straightforward finding was that hospitalized men were consistently older than hospitalized women. This contrasts with reports indicating that men may develop hepatocellular carcinoma at younger ages, with the male-to-female incidence ratio peaking at approximately 50–54 years [43,44]. The discrepancy may reflect differences between cancer incidence and the point at which patients enter hospital care. Older age at first hospitalization could be associated with later diagnosis, a greater burden of comorbidity, differences in referral pathways, or lower engagement with preventive and surveillance services among men [45,46]. Our findings are also consistent with previous clinical observations from north-eastern Poland, where more than 90% of patients with hepatocellular carcinoma were older than 45 years and men predominated. That study also identified cirrhosis, alcohol abuse, and chronic hepatitis B and C infection as the principal underlying risk factors and reported that nearly one-third of patients were ineligible for treatment because of advanced-stage disease at diagnosis [47]. Taken together, these observations suggest that the male excess in hospitalization may arise from both a greater underlying disease burden and sex-related differences in when patients are diagnosed and referred for hospital-based management.

An important finding was the higher hospitalization burden among urban residents. This pattern should not be interpreted as evidence that urban residence itself increases the risk of liver cancer, nor does the present study identify the mechanisms responsible for the difference. Several hypotheses may nevertheless be considered. One possibility is that urban populations differ in their exposure to metabolic risk factors, including obesity, diabetes, and sedentary lifestyles, which are associated with liver cancer development [48,49,50]. Another, non-mutually exclusive hypothesis is that urban residents have greater access to imaging, specialist consultations, tertiary referral centers, and day-hospital procedures, increasing the probability that a liver malignancy is detected, referred, and recorded during hospital care. Conversely, the lower hospitalization rates observed among rural residents could hypothetically reflect underdiagnosis, delayed referral, or reduced access to specialized oncological pathways rather than a genuinely lower occurrence of disease. These interpretations remain speculative because the dataset contained no individual-level information on metabolic risk factors, socioeconomic status, healthcare utilization, diagnostic pathways, travel distance, or disease stage. Therefore, the study cannot determine whether the urban–rural difference reflects variation in underlying disease occurrence, differences in access to diagnosis and referral, differential capture by the hospital system, or a combination of these factors. However, rural–urban disparities in HCC mortality have also been reported, including more pronounced increases in rural populations, partly associated with less favorable declines in hepatitis C virus-related HCC deaths and potentially reduced access to screening and direct-acting antiviral therapies [49,50,51]. Similar disparities have been observed in other cancers and likely reflect complex socioeconomic and healthcare access differences rather than differences in disease occurrence alone [51,52,53]. Accordingly, the potential mechanisms proposed here require further evaluation by linking hospitalization data with cancer registry, outpatient, primary care, socioeconomic, and area-level healthcare access data.

The progressive increase in patient age observed during the study period deserves particular attention. Mean age increased significantly among men, irrespective of residence, and among women living in urban areas. This change is unlikely to have a single explanation. At the population level, it is consistent with the demographic aging of Poland [54]. At the clinical level, improved management of chronic liver diseases may allow more patients with long-standing hepatic injury to survive to ages at which malignant transformation becomes more likely [55,56]. The aging trend is also consistent with the changing etiological structure of liver cancer. While viral hepatitis remains an important cause, the growing contribution of MASLD and alcohol-related liver disease is expected to shift the burden towards older age groups because these conditions generally require decades of progression before malignant transformation occurs [14,57,58,59].

The observed increase in age may therefore reflect a combination of population aging, longer survival with chronic liver disease, and a gradual transition from predominantly infection-related risk toward metabolic and alcohol-related pathways. Nevertheless, because no information on etiology was available, the present study cannot demonstrate that such an etiological transition caused the observed age pattern. The relatively stable length-of-stay distribution over time provides no indication that the increase in patient age was accompanied by a major shift in the duration of hospitalization. However, length of stay is an imperfect proxy for treatment intensity and may be influenced by changes in diagnostic pathways, procedural practices, discharge policies, and the increasing use of day-hospital care. It therefore cannot establish that inpatient management remained unchanged.

Hospitalization rates for hepatocellular carcinoma remained relatively stable in most population groups. In contrast, mean rates for intrahepatic bile duct carcinoma were significantly higher in 2020–2023 than in 2012–2019 across sex and place-of-residence groups. Following the decline observed in 2020, hospitalization rates for intrahepatic bile duct carcinoma increased in subsequent years and, by 2023, exceeded the corresponding pre-pandemic mean rates across most groups. The timing of the initial decline is important because 2020 marked a major disruption to diagnostic and hospital services. The number of first-time C22 hospitalizations decreased by 25.2% between 2019 and 2020, corresponding to 694 fewer hospitalizations than in 2019. This reduction was of similar magnitude to the 22.1% nationwide decrease in the number of patients treated in general-hospital wards, but greater than the 8.1% decrease reported for oncology wards, which corresponded to 28,400 fewer patients [60]. Although oncology ward statistics are not equivalent to diagnosis-based data on hospitalizations for all neoplasms, these national figures provide contextual evidence that the decline observed in our study coincided with a broader contraction in hospital activity. The 2020 decrease should therefore not be interpreted as evidence of an abrupt reduction in the occurrence of liver and intrahepatic bile duct malignancies. It may instead have reflected, at least in part, pandemic-related organizational disruption, changes in healthcare utilization, and reduced access to diagnostic and inpatient pathways. Reduced access to imaging, elective admissions, specialist consultations, and diagnostic procedures may have temporarily lowered the number of malignancies detected or managed in the hospital. The subsequent rise may consequently reflect several overlapping processes, including the restoration of healthcare activity, delayed presentation of cases not diagnosed during the pandemic, changes in referral pathways, and a possible underlying increase in disease occurrence. Nevertheless, the available data do not permit quantification of the relative contributions of healthcare disruption and true epidemiological change.

An increase in intrahepatic cholangiocarcinoma incidence has been reported in several countries, although the underlying reasons remain only partially explained [61,62]. Improved diagnostic classification and advances in imaging undoubtedly contribute, but changing environmental, metabolic, and inflammatory risk factors have also been proposed [62]. These explanations are not mutually exclusive. Greater recognition of intrahepatic cholangiocarcinoma, more precise anatomical classification, and transfer of cases from less specific C22 categories could produce an apparent increase even in the absence of an equivalent change in the total number of primary liver malignancies. Correspondingly, declining hospitalization rates in other C22 categories may partly reflect diagnostic redistribution rather than true reductions in disease incidence [63,64]. The divergent patterns across diagnostic categories should therefore be interpreted jointly rather than as independent trends. Analyses integrating cancer registry, histopathological, mortality, and hospitalization records are needed to determine whether they reflect genuine epidemiological change, improved classification, or both.

Length-of-stay patterns were generally similar between urban and rural residents, suggesting that once hospitalized, patients received comparable inpatient management regardless of place of residence. The lack of major differences may indicate relatively equitable hospital care for liver cancer patients within the Polish healthcare system. However, hospitalization metrics alone cannot assess potential disparities in disease stage at diagnosis, access to specialist treatments, or long-term survival outcomes, all of which would require dedicated investigation. Indeed, it has been previously shown that liver disease in Poland carries a disproportionately high burden of late-stage diagnoses, uneven access to specialized care, and lower long-term survival rates compared to Western European averages [65,66,67]. These findings therefore suggest that any existing inequalities in liver cancer outcomes may arise primarily outside the inpatient setting.

The increasing age of patients and the persistence of substantial disease burden emphasize the importance of prevention. Contemporary evidence suggests that a majority of liver cancer cases could be prevented through effective management of modifiable risk factors [35,67,68]. Although HBV remains a leading global cause of hepatocellular carcinoma, the relative contribution of MASLD, metabolic dysfunction-associated steatohepatitis (MASH), obesity, type 2 diabetes, and alcohol-related liver disease is steadily increasing, particularly in Europe, North America, and Asia [35,68,69]. This shift is also supported by recent nationwide data on first-time HBV hospitalizations in Poland, showing that HBV-related inpatient care is increasingly concentrated in older, multimorbid patients, with age-stratified patterns moving from infection-related conditions in younger groups toward cardiometabolic and advanced hepatic disease in older groups [70]. This epidemiological transition expands the population at risk beyond traditional high-risk groups and presents new challenges for healthcare systems. In Poland, where obesity, diabetes, and alcohol-related morbidity remain important public health concerns [71,72,73], strengthening prevention and diagnostic programs, improving viral hepatitis elimination efforts, and expanding identification of individuals with advanced chronic liver disease may contribute substantially to reducing future liver cancer burden [74]. The findings support a combined strategy rather than a disease-specific approach focused on a single etiology. Such a strategy should include sustained HBV vaccination, identification and treatment of chronic HBV and HCV infection, reduction in harmful alcohol consumption, improved metabolic risk management, and surveillance of patients with cirrhosis or other advanced chronic liver disease.

The findings of this study have several public health implications. The predominance of older male patients suggests that preventive and surveillance strategies should continue to prioritize populations with established liver disease, particularly men with viral, metabolic, or alcohol-related risk factors. At the same time, the increasing age of affected patients and the probable growing contribution of metabolic liver disease support broader integration of liver health assessment into chronic disease prevention programs. Population-level monitoring of hospitalization trends may serve as an important indicator of the effectiveness of these interventions over time.

We would also like to stress several study limitations. First, the analysis was based on administrative hospitalization data rather than clinical records or cancer registry information. Detailed information on disease stage, underlying etiology, laboratory parameters, treatment modalities, and survival outcomes was unavailable. Consequently, we were unable to assess whether changes in hospitalization patterns were associated with differences in diagnostic timing or disease severity at presentation. Second, first-time hospitalization does not necessarily correspond to an incident cancer diagnosis, as some patients may have been diagnosed previously in outpatient settings, while others may never require hospitalization with a C22 diagnosis. Therefore, the study captures a selected subset of patients whose diagnostic or therapeutic pathway involved inpatient or day-hospital care and may underrepresent individuals managed exclusively in outpatient settings. This cohort may differ systematically in disease stage, clinical condition, treatment requirements, or access to care; however, the available administrative data did not permit evaluation of these differences. Consequently, the reported rates should be interpreted as rates of first-time hospitalization rather than estimates of cancer incidence or the characteristics of all newly diagnosed patients. Third, identification of first hospitalizations relied on available demographic characteristics and administrative records, which may have introduced a small degree of misclassification. Fourth, the study included all malignant neoplasms classified in the ICD-10 category C22, a heterogeneous group with varying etiologies and prognoses. Moreover, comorbidities were available only as aggregated ICD-10 chapter-level categories. Consequently, specific etiological liver conditions, including chronic hepatitis B or C and alcohol-related liver disease, could not be identified or analyzed separately. Finally, changes in diagnostic practices, coding accuracy, and healthcare utilization over time may have influenced some of the observed temporal trends. In addition, no counterfactual estimate of the number of hospitalizations expected in 2020 in the absence of the pandemic was constructed, and the study data were not linked to cancer registry records, outpatient diagnostic activity, or diagnosis-based hospitalization data covering all neoplasms. The national statistics used for comparison describe patients treated according to hospital-ward type rather than principal diagnosis and therefore provide contextual evidence of reduced healthcare activity rather than a directly comparable control series. Consequently, although the parallel nationwide decline in hospital activity supports an important organizational contribution, the study cannot definitively distinguish pandemic-related disruption from a genuine change in disease occurrence.

Despite these limitations, the study provides one of the largest nationwide assessments of first-time hospitalizations due to liver and intrahepatic bile duct malignancies in Poland. The long observation period and population-based design enabled the identification of important demographic and geographic patterns that may support future cancer prevention and healthcare planning initiatives.

## 5. Conclusions

First-time hospitalizations due to liver and intrahepatic bile duct malignancies in Poland were characterized by a predominance of men, higher first-time hospitalization rates among urban residents, and a clear age-related pattern, with hospitalizations peaking in the 65–69-year age group. Hepatocellular carcinoma hospitalization rates remained relatively stable in most groups, whereas intrahepatic bile duct carcinoma rates were higher in 2020–2023 than in 2012–2019 and increased after the decline observed in 2020. Because this decline occurred in parallel with a broad nationwide reduction in hospital activity, pandemic-related disruption of diagnostic and hospital pathways may have contributed to it; however, a genuine change in disease occurrence cannot be excluded. These findings warrant continued monitoring, but cannot establish a continuous increase in disease incidence. Given the growing global burden of liver cancer and the substantial proportion of cases that remain preventable, strengthening prevention, early detection, and surveillance strategies should remain a public health priority.

## Figures and Tables

**Table 1:** First-time hospitalizations due to malignant neoplasms of the liver and intrahepatic bile ducts in 2012–2023 in total and by sex and place of residence.

Year	Number of Hospitalizations					First-Time Hospitalization Rates per 100,000 Population					Age-Standardized Hospitalization Rates per 100,000 Population	
	Total	Urban Residents		Rural Residents		Total	Urban Residents		Rural Residents		Total	
		Men	Women	Men	Women		Men	Women	Men	Women	Men	Women
2012	2925	1208	866	486	365	7.6	10.9	7.1	6.4	4.8	12.3	6.5
2013	2545	1010	817	393	325	6.6	9.1	6.7	5.2	4.3	10.2	6.0
2014	2676	1062	823	463	328	7.0	9.6	6.7	6.1	4.3	10.8	5.9
2015	2351	944	687	422	298	6.1	8.6	5.6	5.5	3.9	9.5	5.0
2016	2595	1018	799	442	336	6.8	9.3	6.6	5.8	4.4	10.0	5.6
2017	2777	1145	831	488	313	7.2	10.4	6.8	6.4	4.1	10.8	5.7
2018	2589	1062	749	457	321	6.7	9.7	6.2	6.0	4.2	9.7	5.2
2019	2754	1117	768	496	373	7.2	10.2	6.3	6.5	4.9	10.5	5.5
2020	2060	858	586	355	260	5.4	7.9	4.8	4.6	3.4	7.7	4.0
2021	2350	940	667	432	311	6.2	8.7	5.5	5.7	4.0	8.5	4.6
2022	2468	990	689	492	297	6.5	9.3	5.8	6.5	3.9	9.2	4.6
2023	2808	1087	839	529	353	7.4	10.2	7.1	7.0	4.6	9.8	5.6
2012–2023	30,898	12,441	9121	5455	3880	6.7	9.5	6.3	6.0	4.2	9.9	5.4
	100%	57.7%	42.3%	58.4%	41.6%		*p* < 0.001		*p* < 0.001		*p* < 0.001	

**Table 2:** Age of patients first-time hospitalized due to malignant neoplasms of the liver and intrahepatic bile ducts by place of residence and sex (2012–2023).

	Urban Residents					Rural Residents				
Year	Mean	SD	Q1	Median	Q3	Mean	SD	Q1	Median	Q3
Men										
2012	64.5	14.7	58	66	74	61.0	16.5	55	63	72
2013	65.7	13.4	59	66	75	62.0	16.3	56	64	73
2014	65.6	13.2	59	66	74	63.9	13.4	58	65	72
2015	65.9	13.1	60	66	74	63.8	12.8	57	64	72
2016	65.5	13.8	60	67	74	64.5	13.1	59	65	73
2017	65.8	13.0	60	67	74	63.2	14.1	58	64	71
2018	65.8	12.2	60	66	73	63.7	12.4	58	65	71
2019	67.1	12.9	61	68	75	64.9	12.2	59	66	72
2020	66.2	13.7	60	68	74	62.8	14.9	58	65	71
2021	66.2	12.9	62	68	74	63.3	13.5	58	66	72
2022	65.8	13.1	61	67	74	64.8	12.4	60	67	72
2023	66.3	11.5	60	67	73	65.8	11.1	61	67	72
Mean annual age 2012–2023	*p* for trend < 0.05, R^2^ = 0.42					*p* for trend < 0.05, R^2^ = 0.45				
**Women**										
2012	65.6	16.4	58	68	77	64.2	17.3	56	66	76
2013	68.2	14.2	61	69	78	66.1	15.7	58	67	78
2014	67.4	13.2	60	68	77	66.9	14.1	58	66	78
2015	68.2	13.6	61	69	77	67.3	14.9	59	68	79
2016	68.3	13.8	62	68	78	65.3	15.2	58	66	76
2017	68.2	14.5	62	69	77	65.7	15.3	59	67	75
2018	68.9	13.5	62	70	78	67.8	13.7	61	68	77
2019	69.7	13.9	64	71	79	64.9	16.2	59	67	74
2020	69.1	12.7	64	70	77	66.3	15.4	61	67	75
2021	69.3	11.6	64	70	77	67.3	14.9	61	69	76
2022	67.9	12.8	63	70	75	67.0	14.4	62	69	75
2023	68.5	13.8	63	70	77	67.6	13.1	62	69	75
Mean annual age 2012–2023	*p* for trend < 0.05, R^2^ = 0.38					*p* for trend > 0.05				

SD—standard deviation; Q1—first quartile; Q3—third quartile. *p* for trend was obtained from simple linear regression analysis with calendar year entered as the independent variable.

**Table 3:** Comparison of first-time hospitalization rates due to malignant neoplasms of the liver and intrahepatic bile ducts between the pre-pandemic (2012–2019) and pandemic (2020–2023) periods, according to sex and place of residence.

Year	First-Time Hospitalization Rates per 100,000 Population							
	Men							
	Urban Residents				Rural Residents			
	Liver cell carcinoma (C22.0)	Intrahepatic bile duct carcinoma (C22.1)	Other specified carcinomas of the liver (C22.7)	Other malignant neoplasms (C22.2, C22.3, C22.4, C22.9)	Liver cell carcinoma (C22.0)	Intrahepatic bile duct carcinoma (C22.1)	Other specified carcinomas of the liver (C22.7)	Other malignant neoplasms (C22.2, C22.3, C22.4, C22.9)
Mean annual 2012–2019	4.2	0.8	0.9	3.8	2.2	0.6	0.7	2.6
Mean annual 2020–2023	4.1	1.2	0.6	3.2	2.4	0.7	0.5	2.4
*p*-value	0.33	<0.001	<0.001	<0.001	<0.05	<0.05	<0.001	0.11
	**Women**							
Mean annual 2012–2019	2.0	0.9	0.8	2.9	1.1	0.7	0.6	2.1
Mean annual 2020–2023	1.7	1.2	0.5	2.5	1.0	0.9	0.4	1.8
*p*-value	<0.001	<0.001	<0.001	<0.001	0.18	<0.001	<0.001	<0.005

**Table 4:** First-time hospitalizations due to malignant neoplasms of the liver and intrahepatic bile ducts by place of residence and sex (n, %) in 2012–2023 in length of stay (LOS) classes.

	Urban Residents			Rural Residents			*p*-Value		
	Men								
	LOS 0–3	LOS 4–7	LOS ≥ 8	LOS 0–3	LOS 4–7	LOS ≥ 8	LOS 0–3	LOS 4–7	LOS ≥ 8
Liver cell carcinoma (C22.0)	2184 (40%)	1321 (24%)	1967 (36%)	828 (40%)	491 (24%)	746 (36%)	0.88	0.74	0.89
Intrahepatic bile duct carcinoma (C22.1)	585 (47%)	230 (19%)	418 (34%)	267 (47%)	127 (22%)	178 (31%)	0.76	0.08	0.24
Other specified carcinomas of the liver (C22.7)	281 (26%)	290 (27%)	521 (48%)	138 (24%)	166 (29%)	266 (47%)	0.50	0.27	0.69
Other malignant neoplasms (C22.2, C22.3, C22.4, C22.9)	1591 (33%)	1263 (26%)	1915 (40%)	726 (32%)	661 (29%)	902 (39%)	0.17	<0.05	0.56
	**Women**								
Liver cell carcinoma (C22.0)	1007 (37%)	665 (24%)	1072 (39%)	319 (34%)	225 (24%)	398 (42%)	0.12	0.83	0.09
Intrahepatic bile duct carcinoma (C22.1)	719 (50%)	263 (18%)	442 (31%)	330 (49%)	135 (20%)	209 (31%)	0.52	0.39	0.997
Other specified carcinomas of the liver (C22.7)	220 (22%)	264 (27%)	495 (51%)	119 (25%)	124 (26%)	239 (50%)	0.35	0.61	0.73
Other malignant neoplasms (C22.2, C22.3, C22.4, C22.9)	1310 (32%)	1071 (26%)	1663 (41%)	555 (31%)	496 (27%)	760 (42%)	0.18	0.47	0.54

## Data Availability

The datasets used and analyzed during the current study are available from the corresponding author on reasonable request.
